# Notes and News

**Published:** 1893-10-21

**Authors:** 


### NOTES AND NEWS.

Collected and Communicated.

The entry of medical students at the University of Cambridge this term has reached the unprecedented number of 132.

The new wing of the North Infirmary, Cork, was opened on September 19th. The additional building has been erected as the result of a bequest of £28,000, made a few years ago by the late Lady Combermere, whose father, Dr. Gibbings, was for many years a member of the medical staff of the Infirmary.

Inquiries reach us from time to time concerning the training of ladies as children's nurses. The Norland Institute, 9, Norland Place, Holland Park Gardens, W., undertakes this work, with the twofold object of supplying the public with nurses who have undergone a certain term of training, and of providing employment for educated women who may, from various reasons, be unable to enter upon a longer course of training. Useful and practical teaching is given in matters educational and hygienic, and the scheme of probation extends over a period of nine months. All particulars can be obtained by writing to the Principal, at the Institute.

Mr. F. Lucas's sad death at the Middlesex Hospital adds one more name to the death-roll of medical men who have died martyrs to their duty. Mr. Lucas was casualty physician at the Middlesex Hospital, and during a tracheotomy operation on a child suffering from diphtheria some mucus was thrown on to his face. He completed the administration of the anæsthetic with which he was occupied before removing the danger, in consequence of which he took the infection and fell a victim to the disease. Mr. Lucas had commenced a career of great promise, and his untimely death is deeply regretted by his fellow workers, whose esteem and regard he had won. This life cut short at the threshhold, this devotion to duty in the face of a terrible and well recognised danger, gives an example of heroism far greater than the deeds of valour performed under the exciting influence of a warfare against humanity. But those who fight for humanity against the dangers presented by nature, especially those of disease, do not gain their full popular recognition. Civilisation, however, is preparing a new scroll upon which such names as Mr. Lucas will stand out side by side with those of many a member of the medical profession.

The Bristol Infirmary authorities do not seem overborne by the burden of £5,000 debt. At the recent half-yearly meeting of the Board, the Chairman remarked that the debt had amounted to £10,000 at one time, and practically intimated that it could hardly remain at £5,000, seeing that the yearly difference between income and expenditure averaged about £2,000.

A joint fever hospital at Greenford for West Middlesex districts has been proposed, the site chosen meeting with less opposition than usual. It appears that the usual difficulty which arises as to proportionate rating of the different localities is likely to stand in the way of its erection.

A correspondent to the *Times* writes to suggest that the knowledge acquired by ladies who have attended ambulance classes and obtained a certificate might be usefully applied to the teaching of their poorer neighbours. The writer suggests that this plan should be carried out by holding free lectures in village schools or reading-rooms, and states that such lectures have already been held with success in two villages, and the instruction received has already borne practical fruit.

Amongst the sanitary rules recently enforced in France is one relating to the compulsory destruction of copy and other books belonging to children who have been attacked with infectious diseases. It is now made obligatory that all such things shall be burnt. Certainly most stringent rules are necessary for dealing with every small item as long as the nature and the sources of danger remain so little generally understood. Outward and visible perils are avoidable, and many imaginary risks are shunned. Some people will hesitate to cross the path of an ambulance, although the vehicle may be disinfected and empty, yet when infectious disease declares itself in their own homes they shrink from the destruction of some personal treasure, preferring a possible risk to a definite loss. Until sanitation is commonly taught to all classes of the community there seems but little hope of the complete control of preventable diseases.

Meran, a favourite health resort in the Austrian Tyrol, especially for consumptives, enforces a regulation which might with benefit be adopted in like places elsewhere. We should not expect the measure to be popular with most ladies, as it requires the sacrifice of a certain amount of appearance—at least so some will think. The Meran police have instructions to see that all ladies' dresses clear the ground by some inches, and these instructions are carefully carried out. Offenders are warned, but on the offence being repeated more than twice a fine of £10 is inflicted. Such a procedure on the part of the authorities at Meran will help more effectually towards the adoption of a sanitary and civilised and convenient length of garment by the fair sex than the individual efforts of the few. It may be the case again of the mouse helping the lion, and the small Tyrolean health resort may act as the pioneer in this direction of sanitary education. If Bournemouth and other winter resorts of the consumptive were to set the example, it would doubtless pave the way to the removal of a grave blot on the refinement and civilisation of the women of the day, and tend to check the spread of disease.

